# Modulation of Host Immunity by Microbiome‐Derived Indole‐3‐Propionic Acid and Other Bacterial Metabolites

**DOI:** 10.1002/eji.202451594

**Published:** 2025-04-01

**Authors:** Burkhard Schütz, Felix F. Krause, R. Verena Taudte, Mario M. Zaiss, Maik Luu, Alexander Visekruna

**Affiliations:** ^1^ Institute of Anatomy and Cell Biology Philipps‐University Marburg Marburg Germany; ^2^ Institute for Medical Microbiology and Hygiene Philipps‐University Marburg Marburg Germany; ^3^ Core Facility for Metabolomics Department of Medicine Philipps‐University Marburg Marburg Germany; ^4^ Department of Internal Medicine 3 Rheumatology and Immunology Friedrich‐Alexander‐Universität Erlangen‐Nürnberg (FAU) and Universitätsklinikum Erlangen Erlangen Germany; ^5^ Deutsches Zentrum Immuntherapie (DZI) Friedrich‐Alexander‐Universität Erlangen‐Nürnberg (FAU) and Universitätsklinikum Erlangen Erlangen Germany; ^6^ Lehrstuhl für Zelluläre Immuntherapie, Medizinische Klinik und Poliklinik II Universitätsklinikum Würzburg Würzburg Germany

**Keywords:** gut microbial metabolites, indole‐3‐propionic acid, Intestinal microbiota, mucosal immune system

## Abstract

In recent years, we have witnessed a rapidly growing interest in the intricate communications between intestinal microorganisms and the host immune system. Research on the human microbiome is evolving from merely descriptive and correlative studies to a deeper mechanistic understanding of the bidirectional interactions between gut microbiota and the mucosal immune system. Despite numerous challenges, it has become increasingly evident that an imbalance in gut microbiota composition, known as dysbiosis, is associated with the development and progression of various metabolic, immune, cancer, and neurodegenerative disorders. A growing body of evidence highlights the importance of small molecules produced by intestinal commensal bacteria, collectively referred to as gut microbial metabolites. These metabolites serve as crucial diffusible messengers, translating the microbial language to host cells. This review aims to explore the complex and not yet fully understood molecular mechanisms through which microbiota‐derived metabolites influence the activity of the immune cells and shape immune reactions in the gut and other organs. Specifically, we will discuss recent research that reveals the close relationship between microbial indole‐3‐propionic acid (IPA) and mucosal immunity. Furthermore, we will emphasize the beneficial effects of IPA on intestinal inflammation and discuss its potential clinical implications.

## Graphical abstract

The gut commensal *Clostridium sporogenes* metabolizes dietary tryptophan into indole‐3‐propionic acid (IPA). In this review, we highlight recent findings suggesting that IPA acts as an effective signaling molecule, modulating the function of Th17 lymphocytes and resulting in protection against intestinal inflammation.

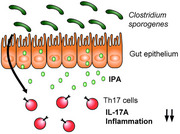

## 1 Introduction

The role of the human microbiome in regulating various aspects of host physiology is now widely well‐recognized. The microorganisms that reside in a symbiotic relationship within the human body offer numerous benefits, particularly by contributing to the maturation of the immune system in early life. They also train the cells of innate and adaptive immunity with long‐term epigenetic effects. Additionally, commensal microorganisms support our health by secreting various immunomodulatory substances and by protecting our body from life‐threatening pathogens [1, 2]. Disruptions in the composition and structure of the human microbiome often lead to gut dysbiosis, which is associated with compromised epithelial integrity, increased intestinal permeability, and chronic inflammation [3]. Elucidating the molecular mechanisms underlying the gut microbiota–host immunity axis could significantly influence the development of next‐generation microbiome‐based therapies. These innovative approaches may eventually complement standard treatments for inflammatory bowel disease (IBD) and autoimmune diseases such as multiple sclerosis and type 1 diabetes. While the composition, diversity, and spatial distribution of the human microbiome across various mucosal tissues are well‐characterized [4], relatively little is known about small molecules and metabolites produced by commensal bacteria, fungi, and other microorganisms. A better characterization of these compounds, many of which are still unknown, will improve our understanding of the close interconnection between the microbiome and host immune responses. Currently, we understand that certain microbiota‐derived factors, such as short‐chain fatty acids (SCFAs), play crucial roles in maintaining immune homeostasis in the gut and other tissues by regulating the balance between regulatory and effector components of the immune system [5]. Furthermore, it has recently become clear that manipulating the composition of microbial communities (e.g., by harnessing the microbiome for particularly interesting members from the stool samples of healthy human individuals) can generate a defined commensal consortium with therapeutic potential. Novel findings suggest that specific commensal communities are able to support antitumor immunity or restore colonization resistance to intestinal pathogens such as multi‐drug resistant nosocomial *Klebsiella* and *Escherichia* species [6, 7]. Such promising commensal candidates might act therapeutically by reshaping the ecological and nutritional niches within the intestinal lumen and by synthesizing bacterial strain‐specific metabolites involved in metabolic competition and pathogen growth inhibition. Notably, a second important class of microbiota‐derived metabolites, secondary bile acids, were shown to inhibit the expansion of *Clostridium difficile* in the gut lumen by suppressing its germination and outgrowth, and by directly binding and neutralizing the TcdB toxin produced by this pathobiont [8, 9] Thus, microbial metabolites produced by commensal bacteria are capable of regulating intestinal homeostasis by creating an immunotolerogenic state in the gut, but also by counteracting immune dysregulation that is linked to pathogen‐driven intestinal inflammation.

### 1.1 Physiological Cross‐Talk between Gut Commensals and the Immune System

In general, intestinal bacteria and their metabolites play a central role in regulating the mucosal immune system. However, there is still a limited understanding of the specific immunomodulatory effects of individual commensal bacterial strains on immune homeostasis. Gut microbiota appear to be essential for the function of colonic regulatory T cells (Tregs) [10]. These CD4^+^ lymphocytes control and suppress aberrant autoimmune reactions, as well as excessive immune responses to food antigens and commensal bacteria [11]. Several gut‐indigenous Clostridia members, as well as *Bacteroides fragilis*, have been shown to promote the differentiation of Foxp3^+^ Tregs in intestinal tissues [12, 13, 14]. Colonizing germ‐free (GF) mice with *B. fragilis, Bacteroides thetaiotamicron*, or certain Clostridia strains, such as *Clostridium immunis* and *Clostridium sporogenes*, not only resulted in the expansion of colonic Tregs but also provided protective effects against dextran sulfate sodium (DSS)‐induced acute colitis [15, 16, 17, 18]. Similarly, the facultative anaerobic commensal *Enterobacter ludwigii* demonstrated its protective capacity in DSS‐induced colitis by triggering dendritic cell (DC)‐mediated Treg polarization, leading to colitis remission [19]. Research focusing on the interaction between microbiota and T cells revealed that the microbiome‐mediated differentiation of Tregs enhances the expression of the nuclear hormone receptor RORγt in these cells [20]. This transcription factor is also critical for regulating the differentiation of IL‐17A‐producing Th17 cells and type 3 innate lymphoid cells (ILCs) [21]. The regulation of maintenance of a delicate balance between tolerogenic immune signals mediated by peripheral Tregs and Th1/Th17 cell‐induced inflammation is a characteristic of many intestinal commensals. For example, the colonization of GF mice with segmented filamentous bacteria (SFB) primarily leads to the generation of Th17 cells in the small intestine [22]. However, in contrast to *Citrobacter rodentium*‐induced pathogenic Th17 responses, Th17 cell population shaped by SFB‐specific antigens appears to have an important homeostatic function in the gut by producing high amounts of anti‐inflammatory cytokine IL‐10 and IL‐22, a factor that supports the integrity of the epithelial barrier [23]. Some intestinal commensals might even be able to promote protective systemic effects in mice. The beneficial effects of *Blautia wexleare* were shown to decrease high‐fat diet‐induced obesity and diabetes by triggering anti‐inflammatory responses and metabolic changes in the host [24]. In summary, gut commensals and their metabolites play a vital role in shaping the functionality of the host immune system, significantly influencing the outcomes of immune‐mediated diseases.

### 1.2 Microbial Metabolites Act as Molecular Messengers, Conveying Biological Signals from the Microbiome to the Host Immune System

The mucosal immune system consists of various effector immune cells, including IgA‐producing plasma cells, macrophages, ILCs, DCs, and T cells [25]. These cells act together to promote tolerance to commensal microorganisms and harmless food proteins while simultaneously combating pathogenic threats. The human intestinal tract provides a natural habitat for a diverse array of bacterial species, each equipped with several gene clusters that encode metabolic enzymes. These enzymatic complexes enable the transformation of dietary polymers, primarily polysaccharides, lipids, and proteins, into a wide range of metabolites. A key feature of commensal bacteria in the colon and cecum is the generation of SCFAs through the anaerobic fermentation of complex, nondigestible polysaccharides, such as dietary fibers. SCFAs represent a major class of gut microbiota‐derived metabolites, playing a pivotal role in communicating with the host immune system by inducing differentiation of Foxp3^+^ Tregs in lamina propria and promoting the production of mucosal IgA antibodies [26, 27, 28, 29]. Notably, the SCFA butyrate has been shown to enhance the production of protective cytokine IL‐22 by modulating the activity of the transcription factors aryl hydrocarbon receptor (AhR) and hypoxia‐inducible factor 1α (HIF1α) [30]. This effect of butyrate appears to be mediated by regulating the activity of mTOR and Stat3. While Stat3 increased the binding of HIF1α to the *Il22* promoter, mTOR activation promoted AhR‐mediated upregulation of IL‐22 production [30]. In addition to SCFAs and branched‐chain fatty acids (BCFAs), various other microbial metabolites, including secondary bile acids, polyamines, microbial tryptophan, indole derivatives, choline metabolites, and vitamins, can traverse the epithelial barrier and influence the function of the mucosal immune system [31]. For instance, the secondary bile acid isoallolithocholic acid (isoalloLCA) and the polyamine spermidine are two other examples of how gut microbial metabolites contribute to intestinal immune homeostasis by promoting polarization and activity of Foxp3^+^ Tregs [32, 33]. Finally, commensal bacteria can convert dietary tryptophan into multiple derivatives that impact the epithelium and underneath immune cells via activation of AhR, pregnane X receptor (PXR), and other cellular factors [34]. It is of substantial clinical interest to better understand if and how these small bacterial molecules can improve the intestinal barrier integrity and anti‐inflammatory immune responses.

### 1.3 Regulation of Immune Responses by Microbial Indole‐3‐Propionic Acid

A large group of microbial metabolites is generated from dietary tryptophan, all of which have the indole scaffold in their core structure. Most tryptophan derivatives produced by commensals serve as ligands for the nuclear receptor AhR, thereby being capable of regulating the integrity of the intestinal barrier, innate immune responses, and overall intestinal homeostasis [35]. Furthermore, by acting as agonists or antagonists for AhR, which is widely expressed in immune cells, these microbial metabolites influence the differentiation of naïve CD4^+^ T cells into Th17 cells and Tregs [36, 37]. Certain strains of the Lactobacillus genus that are capable of metabolizing tryptophan have been shown to alleviate acute colonic inflammation in mice through AhR activation [38]. While common microbial tryptophan catabolites like indole‐3‐lactic acid (ILA) and indole‐3‐acetic acid (IAA) are produced by various commensals, indol‐3‐propionic acid (IPA) is primarily synthesized by one dominant producer, *Clostridium sporogenes*, through reductive Stickland metabolism [39, 40]. The phenyl‐lactate dehydratase gene cluster, including the genes coding for phenyl‐lactate dehydrogenase (*fldH*), phenyl‐lactate dehydratase (*fldBC*), and acyl‐CoA dehydrogenase (*acdA*), seems to be essential for IPA production [39]. Although the capacity to generate IPA from tryptophan is not widespread among gut‐derived commensals, recent studies indicated that smaller amounts of IPA may also be produced by *Peptostreptococcus asaccharolyticus, Peptostreptococcus anaerobius*, and *Clostridium cadaveris* [39, 41]. Several studies have demonstrated that Clostridium species, particularly members of Clostridium clusters I, IV, and XIVa, are abundant in both infants and adults. These bacteria play a crucial role in establishing a tolerogenic environment in the gut by promoting the expression of Foxp3 and facilitating Treg differentiation [13]. Our recent research revealed that GF mice mono‐colonized with *C. sporogenes* significantly increased the production of IPA with approximately 25‐fold higher amounts of this microbial molecule in the colon and serum compared with specific‐pathogen‐free (SPF) mice. Notably, these animals were protected from acute colonic inflammation due to an increase in intestinal IL‐22 secretion, enhanced epithelial barrier integrity, reduction in production of proinflammatory cytokine IL‐17A by T cells, and a higher number of intestinal tuft cells [18].

**FIGURE 1:**
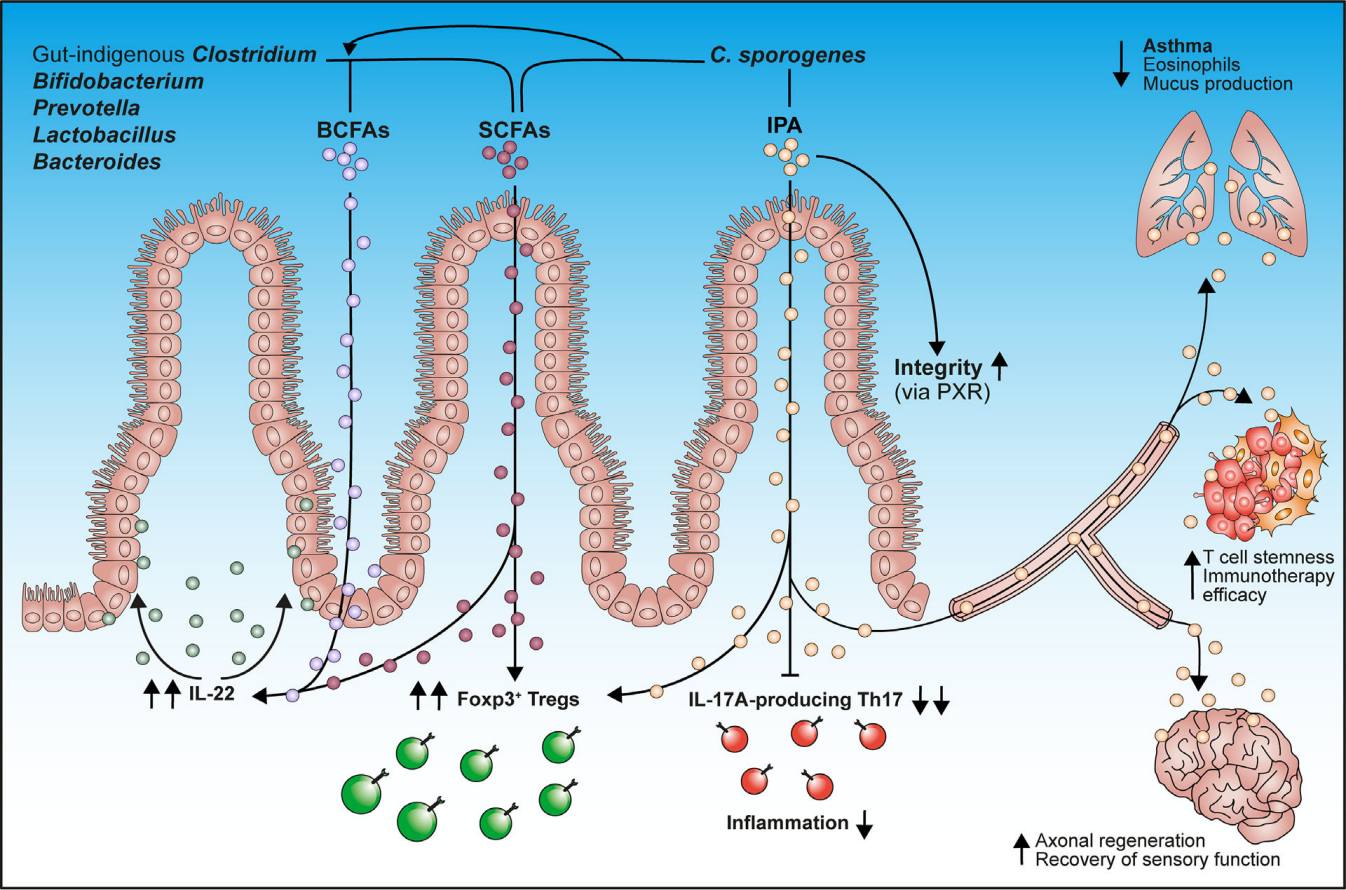
*Clostridium sporogenes*‐derived IPA regulates mucosal and systemic immune responses. The gut microbiota‐derived indole‐3‐propionic acid (IPA) exerts a profound influence on intestinal immune homeostasis, primarily through the regulation of epithelial integrity via its cellular target pregnane X receptor (PXR) and by suppressing the secretion of Th17‐related cytokine IL‐17A during the onset of colitis. In addition, many commensal bacteria produce high amounts of short‐chain fatty acids (SCFAs) and branched‐chain fatty acids that act together with IPA to expand anti‐inflammatory Foxp3^+^ regulatory T cells (Tregs) and increase the production of IL‐22, a protective cytokine that strengthens mucosal barrier integrity. These gut–microbiota‐derived metabolites play an essential role in maintaining intestinal homeostasis. Moreover, the gut‐derived IPA production appears to be involved in early‐life prevention of antibiotic‐induced allergic airway inflammation in mice, in the axonal regeneration and recovery of sensory function, as well as in enhanced immunotherapy efficacy in various preclinical murine tumor models.

Tuft cells are chemosensory cells residing solitary in the epithelia of several mucosal surfaces throughout the body, including the gastrointestinal tract. They are key‐positioned to link luminal cues from the microbiome to the local immune system, thereby, for example, facilitating tissue repair, maintaining epithelial integrity, and combating parasites (helminths, protists, protozoa) [42, 43]. Recently, IPA has been suggested to protect against high‐fat diet‐induced obesity, partly through the activation of tuft cells [44]. In addition to reducing obesity‐related metabolic disturbances and inhibiting gut leakage and inflammation, oral IPA administration led to an increase in the number of tuft cells, both in the small and large intestines. These authors also found in *ex vivo* experiments that the SCFA receptor, Gpr41, is a direct IPA target. Thus, IPA‐mediated activation of tuft cells may trigger the release of multiple effector molecules from this cell type, including acetylcholine, IL25, and leukotrienes, that subsequently modulate local immune reactions. For a long time, the cellular target of IPA on the epithelial and immune cells was elusive. A few years ago, both in vitro and in vivo studies demonstrated that IPA regulates intestinal permeability and inflammation through the orphan nuclear receptor PXR [45]. Moreover, the exposition of intestinal mucosa to *C. sporogenes* was shown to lead to strong induction of the expression of gene encoding IPA's ligand PXR (*Ugt1a1*) [45]. Finally, IPA has been shown to counteract the inflammatory signals triggering the disruption of the epithelial barrier by regulating the expression of epithelial cell stemness and self‐renewal [18]. Collectively, recent studies provide compelling evidence that the microbial metabolite IPA and its cellular target PXR are crucial regulators of intestinal barrier function and intestinal inflammation. The commensal bacterium *C. sporogenes* is also able to produce high concentrations of the BCFAs isobutyrate and isovalerate, which act together with IPA and SCFAs to promote the expansion of colonic Foxp3^+^ Tregs [18]. Furthermore, these bacterial molecules exhibit synergetic effects with regard to the reduction of production of proinflammatory cytokine IL‐17A in the inflamed lamina propria and enhance secretion of colonic protective factors such as IL‐22, ultimately strengthening the intestinal epithelial barrier function (Figure 1).

A recent report has demonstrated that in a mouse model of high salt diet‐induced hypertension, dietary IPA supplementation resulted in decreased systolic blood pressure, along with increased frequency of Tregs and attenuated renal Th17 responses [46]. Interestingly, the serum concentrations of IPA are relatively high under homeostatic conditions (in the micromolar range), raising the intriguing possibility that this microbial molecule may also modulate systemic immune responses. A novel study indicated that IPA generated by *C. sporogenes* can influence the treatment of tumors with immune checkpoint inhibitors and immunotherapy effectiveness in mice [47]. Specifically, in CD8^+^ T cells, IPA increased acetylation levels of H3K27 in a dose‐dependent manner and upregulated the expression of TCF‐1 expression, a factor associated with stem‐like properties promoting tumor control [48]. Moreover, recent research showed that antibody treatment of mice and humans in early life specifically decreased systemic levels of IPA. Importantly, IPA administration to mice prevented exacerbated allergic airway inflammation, the numbers of infiltrated eosinophils, and mitochondrial dysfunction of the lung epithelium during the use of antibiotics in the first year of life [49] (Figure 1). Thus, IPA might be a promising therapeutic candidate for preventing allergic airway inflammation in early life. Additionally, IPA supplementation in mice infected with influenza A virus reduced viral load and ameliorated lung and systemic inflammation [50]. Remarkably, *C. sporogenes*‐derived IPA also promoted nerve regeneration and recovery of sensory function in mice [51]. The bacterial production of IPA seems to promote neutrophil chemotaxis to the dorsal root ganglion–neurons, critically affecting the nerve repair (Figure 1). This study illustrates the ability of gut microbiota‐derived molecules to impact the activity of sensory neurons by modulating the immune system [51]. Together, these findings highlight that microbial tryptophan catabolites, signaling through orphan nuclear receptors such as PXR and AHR, not only regulate intestinal epithelial barrier function locally and mitigate inflammation in the gut but also influence physiological processes systemically in distant tissues.

## Conclusion and Perspectives

There is a growing scientific and clinical interest in gut commensals and their metabolites. Microbial signatures are increasingly recognized for their correlation to therapy responsiveness in various diseases related to immune function. By modifying microbial community composition with beneficial commensals and their metabolites, we can pave the way for personalized interventions in inflammatory diseases such as IBD. This review highlights the recent advances in microbiome research, particularly focusing on the role of microbial metabolite IPA in regulating immune cell function. Of note, microbial BCFAs and SCFAs seem to act synergistically with IPA to support the regeneration process following the disruption of epithelial integrity by inflammation. By illuminating the intricate interactions between gut microbiota‐derived molecules and immune cells in the intestinal compartment, we hope to achieve a deeper understanding of potential therapeutic strategies for modulating local innate and T cell responses in mucosal tissues. We propose a model for immune conditioning in the intestine, where *C. sporogenes*‐derived IPA strengthens the integrity of the epithelial barrier and mitigates proinflammatory T cell responses, ultimately contributing to the suppression of intestinal inflammation.

## Author Contributions

Alexander Visekruna wrote the review. Maik Luu generated the figure. R. Verena Taudte, Mario M. Zaiss, Burkhard Schütz, Maik Luu, and Felix F. Krause performed the final editing. All authors contributed to the article and approved the submitted version.

## Conflicts of Interest

The authors declare no conflicts of interest.

## Data Availability

No new data were generated or analyzed in this study.
